# Magnitude and Associated Factors of Depressive Symptoms Among Adult Patients With Cirrhosis: An Experience From a Resource‐Limited Setting

**DOI:** 10.1155/da/6847420

**Published:** 2026-10-20

**Authors:** Gashahun Mekonnen Dissassa, Geda Lelisa Kube, Henok Fisseha, Eyoel Berhan Mekonnen, Dirbe Jebesa Dinka, Amare Hailu Ashine, Tamirat Godebo Woyimo

**Affiliations:** ^1^ Division of Gastroenterology and Hepatology Jimma University Jimma City Oromia Ethiopia ju.edu.et; ^2^ Division of Gastroenterology and Hepatology SPHMMC Addis Ababa City Addis Ababa Ethiopia; ^3^ SPHMMC School of Public Health Addis Ababa City Addis Ababa Ethiopia; ^4^ Korea Veterans Memorial Health Center Addis Ababa City Addis Ababa Ethiopia; ^5^ Jimma University Jimma City Oromia Ethiopia ju.edu.et

**Keywords:** cirrhosis, depression, Ethiopia, hepatitis B, prevalence, risk factors, social support

## Abstract

**Objective:**

Depression is a common comorbidity in cirrhosis, yet its burden and determinants are poorly characterized in sub‐Saharan Africa. This study aimed to determine the magnitude and factors associated with depression among adult patients with cirrhosis at a tertiary hospital in Ethiopia.

**Methods:**

In a hospital‐based cross‐sectional study, 134 adult patients with cirrhosis were consecutively recruited from a hepatology clinic in Addis Ababa. Depressive symptoms were assessed using the patient health questionnaire‐9 (PHQ‐9), with a score ≥10 indicating clinically significant depressive symptoms. Sociodemographic and clinical data, including cirrhosis etiology, disease stage, MELD score, and social support (3‐item Oslo social support scale [OSS‐3]), were collected via interview and chart review. Multivariable logistic regression was used to identify factors independently associated with clinically significant depressive symptoms.

**Results:**

Twelve of 134 participants (9.0%; 95% CI: 5.3%–15.1%) had clinically significant depressive symptoms based on a PHQ‐9 score ≥10. In adjusted analyses, longer illness duration (adjusted odds ratio [AOR] = 1.48, 95% CI: 1.12–1.96), decompensated cirrhosis was associated with approximately nine‐fold higher odds of clinically significant depressive symptoms compared with compensated cirrhosis, and a higher MELD score (AOR = 1.58, 95% CI: 1.21–2.06) were significantly associated with increased odds of depression. The absence of comorbid chronic illness was also independently associated with lower depression odds (AOR = 0.14, 95% CI: 0.03–0.72, *p* = 0.018). Conversely, moderate (AOR = 0.18, 95% CI: 0.04–0.79) and strong (AOR = 0.04, 95% CI: 0.01–0.64) social support were associated with lower odds of clinically significant depressive symptoms, although the cross‐sectional design precludes conclusions about causality and reverse causation (i.e., depressed individuals may underreport social support) cannot be excluded.

**Conclusion:**

Clinically significant depressive symptoms were identified in 9.0% of adults with cirrhosis. More advanced liver disease and poorer social support were associated with depressive symptoms. These findings underscore the critical need for integrated mental health screening and psychosocial interventions within routine cirrhosis care in resource‐limited settings.

## 1. Introduction

Cirrhosis, the end stage of chronic liver disease, is a major global health problem. Worldwide, cirrhosis accounts for over one million deaths annually and represents one of the leading causes of mortality [1]. In Ethiopia, as in many low‐income countries, the burden is particularly high: liver cirrhosis ranks among the top causes of death and is often linked to viral hepatitis (B and C) and alcohol use [2, 3]. Chronic liver insufficiency not only causes physical complications (ascites, encephalopathy, and bleeding) but can also profoundly affect mental health and quality of life. Depression is a common mental disorder characterized by persistent low mood, loss of interest, and functional impairment [4]. Globally, over 300 million people suffer from depression, which is projected to become the leading cause of disability by 2030 [5]. Depression often co‐occurs with chronic illnesses [6], and patients with long‐term medical conditions are at heightened risk of psychiatric symptoms [7]. It is important to distinguish depressive symptoms identified by screening instruments from depressive disorders established through a formal psychiatric assessment. Screening tools such as the patient health questionnaire‐9 (PHQ‐9) identify individuals with clinically significant depressive symptoms but do not, in isolation, establish a psychiatric diagnosis. In this study, the term depressive symptoms refers specifically to symptoms identified using the PHQ‐9 threshold rather than a clinically diagnosed depressive disorder.

Among patients with cirrhosis, depression is frequently reported but is underrecognized. The prevalence of depressive symptoms in cirrhosis has varied widely in the literature—for example, studies in high‐income settings have found anywhere from ~16% to over 50% of cirrhotic patients scoring positive on depression screens [8–10]. A recent systematic review and meta‐analysis pooling 23 studies comprising 979,113 patients reported a global prevalence of depression in cirrhosis of 37% (95% CI: 29%–46%), with meta‐regression identifying advancing age, alcohol‐related etiology, and viral etiology as significant predictors [11]. Part of this variability reflects disease severity: more advanced liver disease (e.g., decompensation and higher model for end‐stage liver disease [MELD] scores) has consistently been associated with higher depression rates [9]. For instance, one study noted that 24% of patients with early‐stage cirrhosis had mood disorders versus 47% in those with advanced (Child–Pugh C) cirrhosis [9]. Other factors linked to depression in cirrhosis include a longer duration of illness, comorbid medical conditions, and limited social support [10, 12]. Depression in cirrhosis is clinically important because it can worsen the quality of life, impair self‐care and treatment adherence, and even increase mortality [6, 13]. Yet, in many settings, such as Ethiopia, routine psychosocial evaluation of cirrhosis patients is rare.

In Ethiopia and sub‐Saharan Africa more broadly, there is a paucity of data on mental health in liver disease. Despite the known high burden of depression in other chronic diseases locally (for example, heart failure patients in Ethiopia have comorbid depression prevalence >50% [14]), to our knowledge, evidence describing depressive symptoms among Ethiopian adults with cirrhosis remains limited. Given the high prevalence of hepatitis B and the challenges of access to care, Ethiopian patients with cirrhosis may face unique psychosocial stressors. Understanding the magnitude of depression and its correlates in this population is crucial for informing holistic, patient‐centered care.

This study therefore aimed to estimate the prevalence of depressive symptoms and identify associated factors among adult patients with cirrhosis attending a tertiary hepatology clinic in Addis Ababa. We hypothesized that depressive symptoms would be common, particularly among patients with more advanced liver disease or additional comorbidities and that strong social support would be protective. These results are intended to inform holistic clinical practice in resource‐limited settings and highlight the need for psychosocial interventions alongside standard medical treatment.

## 2. Methods

### 2.1. Study Design and Setting

We conducted a hospital‐based analytical cross‐sectional study at Saint Paul’s Hospital Millennium Medical College (SPHMMC) in Addis Ababa, Ethiopia. SPHMMC is a tertiary referral center with a dedicated gastroenterology/hepatology clinic serving patients from across the country. Although the study was conducted in a tertiary referral hospital with access to specialized hepatology services and multidisciplinary clinical care, it is situated within a resource‐limited healthcare system in which access to advanced liver disease management, transplantation, and specialized mental health services remains constrained. Therefore, the cohort represents patients receiving relatively comprehensive care within a broader resource‐limited health system and should not be interpreted as representative of the level of care available to all patients with cirrhosis in Ethiopia. Data were collected over a 4‐month period (January–April 2025).

### 2.2. Eligibility Criteria

All adult patients (aged ≥ 18 years) with a confirmed diagnosis of cirrhosis attending the gastroenterology/hepatology clinic during the study period were eligible. Cirrhosis was defined by clinical, laboratory, imaging, or histological evidence of chronic liver fibrosis with regenerative nodules. Patients were excluded if they had active hepatic encephalopathy (West Haven grade ≥2). Patients were also excluded if they had a documented or previously diagnosed major psychiatric disorder predating the diagnosis of cirrhosis, including major depressive disorder, bipolar disorder, schizophrenia, schizoaffective disorder, or other major psychotic disorders, because these conditions could independently influence depressive symptom scores. A history of depressive symptoms or depressive disorder that developed after the diagnosis of cirrhosis was not an exclusion criterion as depressive symptoms occurring after cirrhosis diagnosis were part of the clinical phenomenon under investigation. Other exclusions included patients who were critically ill and unable to participate or who had hepatocellular carcinoma or other malignancies (due to markedly different prognosis and mental health impact). The aim was to select patients whose depressive symptoms would most likely relate to cirrhosis and its complications.

### 2.3. Operational Definitions

• Depressive symptoms: assessed using the PHQ‐9. Each item was scored from 0 to 3, giving a total score of 0–27. Scores of 5–9, 10–14, 15–19, and 20–27 were categorized as mild, moderate, moderately severe, and severe depressive symptoms, respectively. A total PHQ‐9 score ≥10 was considered indicative of clinically significant depressive symptoms for the primary analysis (25). The PHQ‐9 is a screening instrument and was used in this study to identify clinically significant depressive symptoms; it was not used to establish a diagnosis of depressive disorder. Formal psychiatric diagnosis requires clinical assessment using established diagnostic criteria and, where appropriate, a structured diagnostic interview.
• Social support: measured using the 3‐item Oslo social support scale (OSS‐3). The total score ranges from 3–14, with higher scores indicating stronger support. For analysis, social support was categorized as poor, moderate, or strong according to the standard OSS‐3 cutoffs.
• Cirrhosis was classified as decompensated when there was documented evidence of a cirrhosis‐related decompensating event, including ascites, variceal gastrointestinal bleeding, or overt hepatic encephalopathy. Patients without a documented history of these events were classified as compensated [15]. Compensation status and Child–Pugh class were treated as distinct clinical characteristics. The Child–Pugh class was used to characterize hepatic functional severity, whereas compensation status was determined from the documented history of cirrhosis‐related decompensating events.
• Multimorbidity: defined as the presence of any additional chronic medical illness (such as diabetes, hypertension, and kidney disease) besides cirrhosis. Ascites, variceal bleeding, hepatic encephalopathy, and other cirrhosis‐related complications were not considered comorbidities because they represent manifestations or complications of cirrhosis. The multimorbidity variable included chronic medical conditions independent of cirrhosis, such as diabetes mellitus, hypertension, chronic kidney disease, and other documented chronic diseases.
• MELD score: model for end‐stage liver disease score, a continuous measure of liver disease severity calculated from laboratory values (INR, bilirubin, and creatinine) [16].

### 2.4. Sampling Procedures

This study used a convenience sampling approach based on patient volume at the gastroenterology/hepatology clinic during the 4‐month study period (January–April 2025). During this period, a total of 147 eligible patients with cirrhosis attended the clinic. All were approached for participation, and 134 patients consented to enroll. Given the exploratory nature of this study and the absence of prior local data on depression prevalence in Ethiopian cirrhosis patients on which to base a formal sample size calculation, a convenience sample was deemed appropriate to generate preliminary estimates. The final sample size of 134 provides a 95% confidence interval width of approximately ±5% around a prevalence estimate of 9%, assuming a design effect of 1.0.

### 2.5. Data Collection Procedures and Tools

Data were collected through structured face‐to‐face interviews and a review of medical records. Interviews gathered information on sociodemographic characteristics (age, sex, marital status, education, and residence) and psychosocial factors, including social support (OSS‐3). Clinical data (cirrhosis etiology, duration of diagnosis, presence of complications, MELD and Child–Pugh scores, and coexisting illnesses) were extracted from patient charts and recent investigations. Depression was screened using the PHQ‐9 questionnaire, which has been validated in Ethiopia [14]. Interviewers were trained general practitioners, fluent in local languages (Amharic/Afan Oromo), and questionnaires were pretested to ensure clarity. The PHQ‐9 asks about depressive symptoms over the past 2 weeks; responses are scored 0–3 per item. For quality, PHQ‐9’s internal consistency was checked (Cronbach’s α ~0.86). Data collection sheets were checked daily for completeness by the study supervisors.

Depressive symptoms were assessed using the PHQ‐9 [17]. PHQ‐9 consists of nine items corresponding to DSM‐IV diagnostic criteria, each scored 0–3, yielding a total score of 0–27. We used the Amharic version of PHQ‐9, which has been validated in Ethiopian adult populations [18]. In the validation study, a threshold of ≥10 demonstrated a sensitivity of 84.8% and a specificity of 80.4% for major depressive disorder when compared with structured clinical interview (SCID). For participants preferring Afan Oromo, the questionnaire was administered in that language using the translated version, which underwent forward–backward translation by independent bilingual translators and was pretested for clarity. The internal consistency of PHQ‐9 in our sample was excellent (Cronbach’s *α* = 0.87). For the primary analysis, a total score of ≥10 was used to define clinically significant depressive symptoms, consistent with the locally validated threshold [18]. Scores of 5–9, 10−14, 15–19, and 20–27 were categorized as mild, moderate, moderately severe, and severe depressive symptoms, respectively. PHQ‐9 was administered through face‐to‐face interviews conducted by trained general practitioners who were not part of the clinical care team providing hepatology services to the participants. Interviewers were specifically trained to establish rapport, maintain a neutral demeanor, and reassure participants that their responses would remain confidential and would not influence their clinical care. The questionnaires were administered in a private room to minimize the social desirability bias and ensure confidentiality.

#### 2.5.1. Suicidality Protocol

The PHQ‐9 includes Item 9, which assesses thoughts of self‐harm or suicidal ideation (“thoughts that you would be better off dead or of hurting yourself in some way”). Participants who endorsed Item 9 at any frequency (“several days,” “more than half the days,” or “nearly every day”) were immediately referred to the on‐site psychiatry consultation‐liaison service for further assessment and management. A structured referral pathway was established in collaboration with the Department of Psychiatry at SPHMMC, with a designated psychiatrist available for urgent consultation during clinic hours. Participants with immediate risk were accompanied to the emergency department for emergency psychiatric evaluation. All referrals and outcomes were documented in the medical record. This protocol was reviewed and approved by the Institutional Review Board of SPHMMC (Approval Number: SPHMMC/IRB/23/493/2025).

Social support was measured using the OSS‐3 [19, 20]. OSS‐3 assesses the number of close confidants, sense of concern from others, and practical support availability. Total scores ranged from 3 to 14. Following standard categorization [21], we classified scores of 3–8 as poor social support, 9–11 as moderate social support, and 12–14 as strong social support. The instrument was administered in Amharic and Afan Oromo using locally adapted versions. OSS‐3 has demonstrated acceptable psychometric properties in Ethiopian populations [22, 23].

### 2.6. Data Analysis and Quality Control

Data were entered and analyzed using SPSS version 29. Descriptive statistics (medians and proportions) summarized participant characteristics and depression prevalence. The primary outcome was depression status (PHQ‐9 ≥ 10). The prevalence of clinically significant depressive symptoms was calculated as the proportion of participants with a PHQ‐9 score ≥10. A 95% confidence interval for the prevalence was calculated using the Wilson score method. Bivariate analyses screened for factors associated with depression. Because only 12 participants met the prespecified criterion for clinically significant depressive symptoms, the multivariable model was restricted to a small number of prespecified clinically relevant predictors to minimize overfitting. Potential multicollinearity among candidate predictors was assessed before multivariable modeling using variance‐inflation factors and correlation diagnostics. Variables representing closely overlapping aspects of liver disease severity were not simultaneously included in the final model. For continuous predictors, the linearity of the association with the log odds of clinically significant depressive symptoms was assessed before fitting the final model. Adjusted odds ratios (AORs) with 95% confidence intervals were reported; *p* < 0.05 was considered statistically significant. Data quality measures included interviewer training, questionnaire pretesting, daily review of interviews, and checking for data entry errors. Imputation methods were not needed as the dataset was complete for key variables. The study protocol conformed to ethical standards, and all participants provided informed consent before enrollment.

## 3. Results

### 3.1. Participant Flow

During the study period (January–April 2025), a total of 147 adult patients with confirmed cirrhosis attended the gastroenterology/hepatology clinic at SPHMMC. Of these, 13 (8.8%) were excluded for the following reasons: six (4.1%) had active hepatic encephalopathy (West Haven grade ≥2), four (2.7%) had hepatocellular carcinoma, two (1.4%) had a documented major psychiatric disorder predating cirrhosis, and one (0.7%) was critically ill and unable to participate. No patient declined participation. The remaining 134 patients (91.2%) were enrolled in the study and included in the final analysis.

### 3.2. Sociodemographic Profile

The median age of the 134 cirrhotic patients was 42 years (IQR: 32–52), with ages ranging from 18 to 72 years. Females comprised the majority, 80 (59.7%), while males comprised 54 (40.3%). The largest age groups were 28–37 years (44; 32.8%) and 38–47 years (39; 29.1%). Regarding education, most patients had completed secondary school (53; 39.5% Table 1).

**Table 1:** Sociodemographic characteristics of cirrhotic patients on follow‐up at gastroenterology and hepatology clinics of SPHMMC, Addis Ababa 2025 (*n* = 134).

Variable	Category	Frequency (*n*)	Percentage (%)
Sex	1. Male	54	40.3
	2. Female	80	59.7
Age	1. 18–27 years	9	6.7
	2. 28–37 years	44	32.8
	3. 38–47 years	39	29.1
	4. 48–57 years	27	20.2
	5. 58–67 years	9	6.7
	6. 68 and above years	6	4.5
Educational status	1. Elementary	36	26.9
	2. Secondary	53	39.5
	3. Certificate, diploma, degree	32	23.9
	4. No formal education	13	9.7
Marital status	1. Single	37	27.6
	2. Married	84	62.7
	3. Divorced	6	4.5
	4. Widowed	7	5.2
Residency	1. Urban	103	76.8
	2. Rural	31	23.2
Employment	1. Government employee	24	17.9
	2. Self employed	43	32.1
	3. Unemployed	23	17.1
	4. Housewife	30	22.4
	5. Retired	14	10.4

### 3.3. Clinical, Behavioral, and Individual Characteristics

Overall, 15 (11.2%) of patients reported a history of alcohol use, five (3.7%) were current cigarette smokers, and six (4.5%) had a khat‐chewing habit. The mean (±SD) duration since diagnosis was 4.8 (±2.1) years. Comorbid conditions were present in 33 (24.6%) patients. Among these, the most frequent comorbidities were diabetes mellitus in eight patients (5.97%), hypertension in seven patients (5.22%), chronic kidney disease in two patients (1.49%), and other documented chronic conditions in 16 patients (11.94%). Ascites, variceal bleeding, hepatic encephalopathy, and other cirrhosis‐related complications were not classified as comorbidities as they represent manifestations of cirrhosis itself (Table 2).

**Table 2:** Clinical, behavioral, and individual characteristics of cirrhotic patients on follow‐up at gastroenterology and hepatology clinics of SPHMMC, Addis Ababa 2025 (*n* = 134).

Variables	Category	Frequency (*n*)	Percentage (%)
History of alcohol abuse	Yes	15	11.2
Cigarette smoking	Yes	5	3.7
Khat‐chewing habit	Yes	6	4.5
Comorbidities	Yes	33	24.6
Cirrhosis‐related complications			
Presence of Ascites	Yes	20	15
Upper GI bleeding the previous month	Yes	6	4.5
Hepatic encephalopathy in the previous month	Yes	6	4.5

### 3.4. Cirrhosis‐Specific Risk Factors

#### 3.4.1. Etiologies of Cirrhosis

Hepatitis B was the predominant etiology (73.13%), followed by Hepatitis C (14.20%). Alcohol‐related liver disease accounted for 5.97%. (Figure 1)

**Figure 1:**
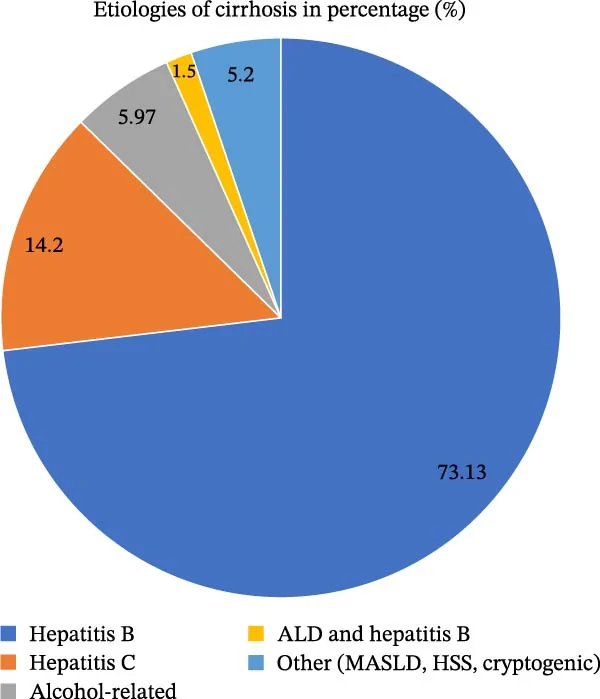
Etiological distribution of cirrhosis among study participants on follow‐up at gastroenterology and hepatology clinics of SPHMMC, Addis Ababa 2025 (*n* = 134).

Two‐thirds of patients had compensated cirrhosis, 89 (66.4%), while 45 (33.6%) were decompensated. Complications of cirrhosis were present in 63 (47.0%) patients, and 21 (15.7%) had been hospitalized for a liver‐related illness in the preceding 6 months. Ten patients (7.5%) had clinical indications for liver transplantation, but none had undergone liver transplantation. By Child–Pugh classification, the majority were Class A 89 (66.4%) (Table 3).

**Table 3:** Cirrhosis specific characteristics and social support among cirrhotic patients on follow‐up at gastroenterology and hepatology clinics of SPHMMC, Addis Ababa 2025 (*n* = 134).

Variables	Category	Frequency (*n*)	Percentage (%)
Type of cirrhosis	Compensated	89	66.4
	Decompensated	45	33.6
Presence of complication of cirrhosis	Yes	63	47.0
Hospitalization for liver related disease in last 6 months	Yes	21	15.7
Liver transplant requirement	Yes	10	7.5
Child–Pugh class	Child Class A	89	66.4
	Child Class B	39	29.1
	Child Class C	6	4.5
Oslo social support	Poor social support	22	16.4
	Moderate social support	89	66.4
	Strong social support	23	17.2

### 3.5. Prevalence of Depression Among Cirrhotic Patients

Among the 134 participants, 7 (5.2%) had mild depressive symptoms, five (3.7%) had moderate depressive symptoms, seven (5.2%) had moderately severe depressive symptoms, and one (0.7%) had minimal symptoms. Based on the prespecified PHQ‐9 threshold of ≥ 10, 12 participants 9.0% (95% CI: 5.3%–15.1%), had clinically significant depressive symptoms. No participant had a PHQ‐9 score in the severe range (20–27) (Figure 2).

**Figure 2:**
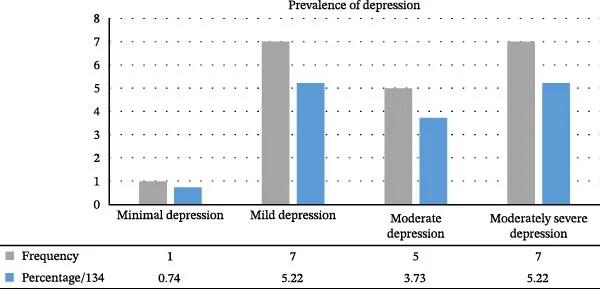
Distribution of PHQ‐9 depressive symptom severity among adults with cirrhosis (*n* = 134).

#### 3.5.1. Factors Associated With Depression

In the multivariable logistic regression model (Table 4), which was limited to prespecified predictors to avoid overfitting given the 12 outcome events, decompensated cirrhosis was associated with approximately nine‐fold higher odds of clinically significant depressive symptoms compared with compensated cirrhosis. Higher MELD score was also independently associated with increased depression odds (AOR = 1.58, 95% CI: 1.21–2.06, *p* = 0.001). Moderate and strong social support were independently protective (AOR = 0.18, 95% CI: 0.04–0.79, *p* = 0.023 and AOR = 0.04, 95% CI: 0.01–0.64, *p* = 0.023, respectively). The absence of comorbid chronic illness was also independently associated with lower depression odds (AOR = 0.14, 95% CI: 0.03–0.72, *p* = 0.018). The model showed an apparent AUC of 0.84 (95% CI: 0.76–0.92).

**Table 4:** Bivariate and multivariable analyses of factors associated with clinically significant depressive symptoms (PHQ‐9 ≥ 10) among adults with cirrhosis at SPHMMC, Addis Ababa, 2025 (*n* = 134).

Variable	Category	Depression (PHQ‐9 ≥ 10)		COR (95% CI)	AOR (95% CI)	*p*‐Value
		Yes (*n* = 12)	No (*n* = 122)			
Disease severity						
Type of cirrhosis	Compensated	3 (25.0%)	86 (70.5%)	0.08 (0.02–0.29)	**0.11 (0.02–0.58)**	**0.009**
	Decompensated	9 (75.0%)	36 (29.5%)	1 (ref)	1 (ref)	—
MELD score	Mean ± SD	18.2 ± 5.4	12.6 ± 4.1	1.52 (1.18–1.96)	**1.58 (1.21–2.06)**	**0.001**
Social support						
Oslo social support scale	Poor	5 (41.7%)	17 (13.9%)	1 (ref)	1 (ref)	—
	Moderate	6 (50.0%)	83 (68.0%)	0.25 (0.07–0.85)	**0.18 (0.04–0.79)**	**0.023**
	Strong	1 (8.3%)	22 (18.1%)	0.06 (0.01–0.78)	**0.04 (0.01–0.64)**	**0.023**
Comorbidity						
Presence of other Chronic illness	Yes	7 (58.3%)	26 (21.3%)	1 (ref)	1 (ref)	—
	No	5 (41.7%)	96 (78.7%)	0.19 (0.06–0.65)	**0.14 (0.03–0.72)**	**0.018**

*Note:* AORs were estimated using Firth penalized logistic regression because of the small number of outcome events. Bold values show the significant variables in both the AOR and their respective *p*‐values.

### 3.6. Sensitivity Analysis of PHQ‐9 Cognitive‐Affective Items

To address potential confounding by somatic symptoms of cirrhosis, we conducted a sensitivity analysis restricted to the cognitive and affective items of the PHQ‐9 (items 1, 2, 6, 7, 8, and 9: anhedonia, depressed mood, guilt/worthlessness, concentration difficulties, psychomotor changes, and suicidal ideation). When applying a threshold of ≥5 on these six items (equivalent to the ≥10 threshold on the full scale), the prevalence of clinically significant depressive symptoms was 7.5% (10/134, 95% CI: 4.1%–13.2%). The association with decompensated cirrhosis remained significant (AOR = 7.82, 95% CI: 1.42–43.1), while the association with the MELD score was attenuated (AOR = 1.32, 95% CI: 0.98–1.78).

## 4. Discussion

In this first investigation of depression among Ethiopian patients with cirrhosis, the 9.0% prevalence observed in our cohort is substantially lower than the pooled global estimate of 37% from a recent meta‐analysis [11]. This discrepancy likely reflects several factors. First, our cohort was predominantly composed of patients with compensated cirrhosis (66.4%) and hepatitis B as the primary etiology (73.1%), whereas the meta‐analysis included a higher proportion of patients with alcohol‐related liver disease and decompensated cirrhosis, both of which were identified as predictors of depression. Second, our study was conducted in a resource‐limited setting, where patients may underreport psychological symptoms due to cultural factors or limited mental health literacy. Third, the meta‐analysis included studies using varied screening instruments and thresholds, whereas our use of the locally validated PHQ‐9 threshold of ≥10 may have yielded more conservative estimates.

Selection effects may also have contributed substantially to the relatively low prevalence observed in our cohort. Patients with hepatocellular carcinoma or other malignancies, overt hepatic encephalopathy, and those who were critically ill or unable to participate were excluded, although these groups may have substantial psychological and psychiatric vulnerability. In addition, participants were recruited from a single tertiary hepatology outpatient clinic, and therefore, the study population may differ from hospitalized patients, patients receiving care in less specialized settings, and individuals with limited access to specialist care. These design‐related factors should be considered when comparing our prevalence estimate with those in international studies. Thus, the relatively low prevalence observed in this study should not be attributed solely to the predominance of hepatitis B or other etiological differences. In contrast, cohorts enriched for alcohol‐related cirrhosis or decompensated disease tend to show far higher psychiatric comorbidity [8, 9]. The wide range reported in the literature underscores that cirrhosis is a heterogeneous condition: when comparing studies, one must consider factors like etiology, disease stage, and cultural context.

We observed that markers of more advanced liver disease strongly predicted depression. A longer duration of illness, decompensation (ascites, encephalopathy, etc.), and higher MELD score all increased the odds of clinically significant depressive symptoms. This pattern is consistent with the prior research. Other studies have similarly noted that fatigue, hospitalizations, and the physical toll of decompensated cirrhosis amplify psychological stress [12, 24]. The biological consequences of liver failure may contribute as well: chronic inflammation and hyperammonemia in cirrhosis can affect neurochemistry and mood [25]. Thus, the strong link we found between disease severity and depression aligns with a growing consensus that cirrhosis itself is significantly associated with psychiatric symptoms [13, 26]. Clinically, this implies that patients with more severe cirrhosis—identified by complications or high MELD—warrant proactive mental health screening. Waiting until patients become symptomatic may miss a window when psychosocial support could prevent deterioration.

Comorbid chronic illnesses also emerged as an independent factor associated with clinically significant depressive symptoms. In our sample, patients with any additional medical conditions (e.g., diabetes, hypertension, and chronic kidney disease) had higher depression rates. This is logical: multimorbidity increases the overall disease burden and complexity of care, leading to more stress and functional impairment [27]. Indeed, the presence of another chronic illness likely compounds the psychological impact of cirrhosis. Similar associations have been reported in other settings, where heart failure or diabetes plus cirrhosis heighten depression risk [14, 27, 28]. In practical terms, clinicians should be alert to the mental health needs of cirrhosis patients with additional diagnoses. Comprehensive care for these individuals should explicitly address the cumulative psychosocial stress of managing multiple illnesses.

We found a strong statistical association between social support and lower odds of clinically significant depressive symptoms. While this finding is consistent with the established literature showing that social support can buffer stress and promote coping [29, 30], it is equally plausible that the direction of causation operates in the opposite direction: individuals with depressive symptoms may withdraw from social relationships and perceive available support more negatively, leading to lower reported social support [31, 32]. The cross‐sectional design of this study precludes determining whether social support protects against depression, whether depression erodes social support, or both. This finding resonates with a large body of evidence in chronic disease: robust family and community support buffers stress, promotes coping, and reduces the risk of depression [10, 33]. In the context of cirrhosis, social support may encourage adherence to medical regimens, assist with clinic attendance, and provide emotional comfort during hospitalizations—all of which can mitigate mood symptoms. A prior study even linked adequate family support to improved survival in end‐stage liver disease [18]. Our results suggest that psychosocial factors such as support networks are pivotal determinants of mental health in cirrhosis, perhaps even more amenable to intervention in a resource‐limited setting than biological factors. Simple measures—like involving the family in counseling or connecting patients with support groups—could have a large impact.

In high‐income countries, many cirrhosis studies focus on transplant candidates or hospitalized patients, leading to higher reported depression. Our prevalence aligns more closely with some general outpatient studies. It is also worth noting that depression in cirrhosis shares features with depression in other chronic conditions: for example, Ethiopian studies of heart failure [14] and diabetes [16] have reported depression prevalences of 40%–60%. The fact that our cirrhosis cohort was lower suggests again that disease characteristics (and perhaps under‐recognition) play a role. Regardless, the comparison highlights that depression is a widespread problem in chronic illness in Ethiopia, and hepatology patients are a part of this pattern.

A key implication of our findings is the need for a holistic care approach. Often, in hepatology clinics, the focus is on managing ascites, varices, or liver transplant preparation. However, our data show that the “mind” of the patient cannot be ignored: approximately one in 11 patients met the prespecified criterion for clinically significant depressive symptoms. Depression can undermine self‐care and adherence [14] and exacerbate feelings of hopelessness that may deter patients from seeking care. Therefore, liver clinics—even in low‐resource settings—should incorporate mental health screening. PHQ‐9 is brief and has been validated locally [18], making it feasible for routine use. Patients with clinically significant depressive symptoms should receive further clinical assessment and appropriate management, including psychosocial interventions, family or peer support, and referral for specialized mental health care when indicated. Pharmacological treatment with antidepressants may also be considered for patients with clinically diagnosed depressive disorders according to clinical assessment and treatment guidelines.

Moreover, these results emphasize that treating cirrhosis effectively requires addressing the psychosocial domain in addition to the organic pathology. An integrated care model is advocated: multidisciplinary teams that include counselors or social workers may be ideal, but at minimum, hepatologists and nurses should receive training to recognize psychological distress. In practice, this could mean asking about mood and support during clinic visits and mobilizing community or family resources to support patients. Because strong social support was so protective, interventions like family education, community health volunteers, or support groups for patients with liver disease could be very beneficial and may be cost‐effective in our setting.

Our study also highlights a broader point: managing a chronic condition like cirrhosis is not solely a matter of prescribing medication or procedures. Emotional, social, and economic factors can profoundly influence health outcomes. In Ethiopia’s resource‐limited environment, biological therapies for advanced liver disease (e.g., transplantation) are scarce, so leveraging psychosocial strengths is even more crucial. By addressing depression and enhancing support, we may improve patients’ quality of life and possibly their clinical trajectory.

Overall, clinically significant depressive symptoms were identified in 9.0% of adults with cirrhosis in this Ethiopian tertiary hepatology cohort. Depressive symptoms were more strongly associated with greater disease severity, including decompensated cirrhosis, higher MELD scores, and longer duration of illness, whereas moderate or strong social support was associated with lower odds of depressive symptoms. However, these findings should be interpreted in light of the cross‐sectional design, single‐center recruitment, and exclusion of patients with hepatocellular carcinoma, overt hepatic encephalopathy, and those unable to participate, which may have contributed to the relatively low prevalence observed. Although the findings support consideration of depression screening and psychosocial assessment within cirrhosis care, larger multicenter studies with broader patient inclusion and assessment of both depression and anxiety are needed to better characterize the psychological burden of cirrhosis in Ethiopia.

## 5. Strengths and Limitations

This study provides preliminary evidence on clinically significant depressive symptoms among adults with cirrhosis in Ethiopia. Strengths include the use of validated screening and social support instruments, detailed clinical characterization including MELD and Child–Pugh scores, and multivariable analysis to identify factors independently associated with depressive symptoms.

Several limitations should be considered. First, the cross‐sectional design precludes causal inference. Second, consecutive recruitment from a single tertiary hepatology clinic may have introduced selection bias and limited generalizability to patients receiving care in primary or secondary settings or those without access to specialized care. Third, the PHQ‐9 was administered through face‐to‐face interviews by trained general practitioners who were not part of the clinical care team, which may have introduced a social desirability bias. In Ethiopian settings, where mental health stigma remains prevalent, patients may underreport depressive symptoms in face‐to‐face interviews due to the fear of labeling or discrimination [34, 35]. This mode of administration likely contributed to the relatively low prevalence observed (9.0%) compared to self‐administered screening or diagnostic interviews in other settings. Future studies should consider using self‐administered instruments or computer‐assisted methods to minimize the social desirability bias. Fourth, depressive symptoms were assessed using the PHQ‐9 rather than a structured psychiatric interview; therefore, a PHQ‐9 score ≥10 indicates clinically significant depressive symptoms and does not establish a depressive disorder. Some PHQ‐9 somatic symptoms may also overlap with the manifestations of cirrhosis. Although the PHQ‐9 was selected because it is brief, widely used, and validated for screening depressive symptoms, it includes somatic symptoms such as fatigue and sleep disturbance that may overlap with manifestations of cirrhosis. Other screening instruments, such as the hospital anxiety and depression scale (HADS), place less emphasis on somatic symptoms and may therefore provide complementary assessment in medically ill populations. This potential overlap may have resulted in some misclassification of depressive symptoms. In addition, the association between social support and depressive symptoms must be interpreted with caution. Finally, although patients with known major psychiatric disorders and overt hepatic encephalopathy were excluded, psychiatric history was not systematically assessed, and minimal hepatic encephalopathy was not formally evaluated. Unrecognized psychiatric conditions or minimal hepatic encephalopathy may therefore have contributed to the observed depressive symptoms. Minimal hepatic encephalopathy can require specialized psychometric or neurophysiological testing for detection. Antidepressant medication use was not systematically assessed or recorded, and therefore, its potential influence on depressive symptom scores could not be evaluated. Despite these limitations, the findings provide useful preliminary data and support the need for larger multicenter studies incorporating structured psychiatric assessment and standardized evaluation of minimal hepatic encephalopathy.

## 6. Conclusion

Clinically significant depressive symptoms were identified in 9.0% of adults with cirrhosis in this tertiary Ethiopian clinic. Advanced liver disease (as indicated by decompensation, higher MELD scores, and longer illness duration) and additional chronic illnesses were also significantly associated with higher odds of clinically significant depressive symptoms. In contrast, patients with moderate or strong social support had substantially lower odds of clinically significant depressive symptoms. These results demonstrate that, even in a resource‐limited setting, mental health is a key component of care for cirrhosis patients. We recommend routine screening for depression (using a simple tool such as the PHQ‐9) as part of standard hepatology practice. Management should address not only the physical complications of cirrhosis but also the psychological and social needs. In particular, interventions that build family and community support could mitigate the high risk of depression in this population. Finally, these findings call for a shift toward holistic, patient‐centered care: multidisciplinary teams should include mental health resources, and care models should integrate psychosocial support alongside medical treatment. Such approaches may improve adherence, quality of life, and possibly medical outcomes for patients living with cirrhosis in Ethiopia and similar settings.

## Author Contributions

Conceptualization, methodology, investigation, visualization: Gashahun Mekonnen Dissassa, Geda Lelisa Kube, Henok Fisseha, Eyoel Berhan Mekonnen, and Tamirat Godebo Woyimo. Software: Gashahun Mekonnen Dissassa. Validation: Geda Lelisa Kube, Henok Fisseha, and Eyoel Berhan Mekonnen. Formal analysis: Gashahun Mekonnen Dissassa, Tamirat Godebo Woyimo, and Eyoel Berhan Mekonnen. Resources: Gashahun Mekonnen Dissassa and Amare Hailu Ashine. Data curation: Gashahun Mekonnen Dissassa, Geda Lelisa Kube, Henok Fisseha, Eyoel Berhan Mekonnen, Dirbe Jebesa Dinka, and Tamirat Godebo Woyimo. Writing – original draft preparation, writing – review and editing: Gashahun Mekonnen Dissassa, Geda Lelisa Kube, Henok Fisseha, Eyoel Berhan Mekonnen, Dirbe Jebesa Dinka, Amare Hailu Ashine, and Tamirat Godebo Woyimo. Supervision: Geda Lelisa Kube, Henok Fisseha, and Eyoel Berhan Mekonnen.

## Funding

No funding was received for this manuscript.

## Disclosure

All authors agreed to bear responsibility for all parts of the research work and approved the final version of the manuscript. No third‐party services or persons beyond the authors were involved in the research or manuscript preparation.

## Ethics Statement

Ethical clearance was obtained from the Institutional Review Board (IRB) of St. Paul’s Hospital Millennium Medical College (SPHMMC) (Approval Number: SPHMMC/IRB/23/493/2025). Patient confidentiality was strictly maintained throughout the study, with only the principal investigator, supervisor, and data collectors having access to patient information. This study was conducted in accordance with the guidelines of Good Clinical Practice and the Principles of the Declaration of Helsinki. Written informed consent was obtained from all participants prior to their enrollment in the study.

## Consent

The authors have nothing to report.

## Conflicts of Interest

The authors declare no conflicts of interest.

## Data Availability

The deidentified participant‐level dataset, data dictionary, and SPSS syntax used for the analyses reported in this study are available from the corresponding author upon reasonable request, subject to appropriate institutional and ethical requirements. Requests for access may be directed to the corresponding author. All data were kept confidential and used solely for research purposes.
